# Pyramiding of Fusarium Head Blight Resistance Quantitative Trait Loci, *Fhb1, Fhb4*, and *Fhb5*, in Modern Chinese Wheat Cultivars

**DOI:** 10.3389/fpls.2021.694023

**Published:** 2021-07-14

**Authors:** Yiduo Zhang, Zibo Yang, Haicai Ma, Liying Huang, Feng Ding, Yingying Du, Haiyan Jia, Guoqiang Li, Zhongxin Kong, Congfu Ran, Zhengzhong Gu, Zhengqiang Ma

**Affiliations:** ^1^Crop Genomics and Bioinformatics Center, Nanjing Agricultural University, Nanjing, China; ^2^National Key Laboratory of Crop Genetics and Germplasm Enhancement, Nanjing Agricultural University, Nanjing, China; ^3^Huaiyin Institute of Agriculture Sciences of Xuhuai Region in Jiangsu, Huaian, China

**Keywords:** wheat, Fusarium head blight, marker-assisted selection, gene pyramiding, *Fhb1*, *Fhb4*, *Fhb5*

## Abstract

Wheat production is increasingly threatened by the fungal disease, Fusarium head blight (FHB), caused by *Fusarium* spp. The introduction of resistant varieties is considered to be an effective measure for containment of this disease. Mapping of FHB-resistance quantitative trait locus (QTL) has promoted marker-assisted breeding for FHB resistance, which has been difficult through traditional breeding due to paucity of resistance genes and quantitative nature of the resistance. The lab of Ma previously cloned *Fhb1*, which inhibits FHB spread within spikes, and fine mapped *Fhb4* and *Fhb5*, which condition resistance to initial infection of *Fusarium* spp., from FHB-resistant indigenous line Wangshuibai (WSB). In this study, these three QTLs were simultaneously introduced into five modern Chinese wheat cultivars or lines with different ecological adaptations through marker-assisted backcross in early generations. A total of 14 introgression lines were obtained. All these lines showed significantly improved resistance to the fungal infection and disease spread in 2-year field trials after artificial inoculation. In comparison with the respective recipient lines, the *Fhb1, Fhb4*, and *Fhb5* pyramiding could reduce the disease severity by 95% and did not systematically affect plant height, productive tiller number, kernel number per spike, thousand grain weight, flowering time, and unit yield (without *Fusarium* inoculation). These results indicated the great value of FHB-resistance QTLs *Fhb1, Fhb4*, and *Fhb5* derived from WSB, and the feasibility and effectiveness of early generation selection for FHB resistance solely based on linked molecular markers.

## Introduction

Fusarium head blight (FHB) or scab is a global fungal disease in wheat caused by *Fusarium* spp., particularly *Fusarium graminearum* Schwabe (teleomorph: *Gibberella zeae*) (Ma et al., 2020). Apart from reducing yield and deteriorating grain quality, the pathogen produces mycotoxins, such as deoxynivalenol (DON), in kernels that are harmful to human and livestock health (Gilbert and Tekauz, 2000). In China, wheat FHB epidemics frequently occur in the middle and lower reaches of the Yangtze River and the south of the Huang-Huai area, where the flowering stage of wheat often meets with a warm and humid environment. However, due to global warming and changes in cultivation practices in recent years, FHB occurrence has become more and more frequent in the north and west of China. Of the measures that could be taken to control FHB (Chen et al., 2019; Ma et al., 2020), deployment of FHB-resistant cultivars is fundamental and favored by farmers for its environmental friendliness and cost-effectiveness.

Fusarium head blight resistance is a quantitative trait controlled by polygenes and greatly affected by the environment. Making matters more complicated is that it could take different forms, for instance, type I resistance (against initial infection), type II resistance (against fungal spread within the spike), type III resistance (low toxin accumulation in kernels), type IV resistance (lower kernel infection rate), and type V resistance (host tolerance) (Schroeder and Christensen, 1963; Miller et al., 1985; Mesterházy, 1995). These factors pose great difficulties on phenotype evaluation because of the requirement for suitable facilities, different inoculation methods and assessments, repeated trials, and considerable labor and time investment, and thus limit the efficiency of FHB resistance improvement through conventional breeding. The advent of marker-assisted selection (MAS) provides a very promising option to overcome these problems (Dudley, 1993; Lee, 1995; Miedaner et al., 2006; Buerstmayr et al., 2009; Xue et al., 2010a; Nayak et al., 2017; Jia et al., 2018; Ma et al., 2020). Until now, more than 432 quantitative trait loci (QTLs) for FHB resistance have been mapped in wheat (Ma et al., 2020), of which many for type I and type II resistances overlap with QTLs for other types of resistance, indicating the principal roles of type I and type II resistances in controlling FHB. Some of these QTLs have been applied to MAS-based FHB resistance improvement with success (Miedaner et al., 2006; Buerstmayr et al., 2009; Xue et al., 2010a; Salameh et al., 2011; Bernardo et al., 2013; Zhang et al., 2016; Jia et al., 2018; Brar et al., 2019a; Li et al., 2019a); however, most of them still require verification due to small effects and large confidence intervals.

No accessions or lines showing immunity to FHB have been found among wheat germplasm. In wheat breeding programs worldwide, FHB-resistant Sumai 3, a wheat cultivar developed from the cross of Funo with Taiwanxiaomai by Suzhou Institute of Agricultural Sciences, China, and its derivatives are the main sources of FHB resistance (Ban and Suenaga, 2000; Buerstmayr et al., 2003; Frohberg et al., 2006; Marza et al., 2006; Badea et al., 2008; Anderson et al., 2012, 2019; Bernardo et al., 2013; Li et al., 2019c). The utilization of Sumai 3-derived resistance genes has only been partially successful so far because of the difficulty in simultaneous improvement of the resistance and agronomic traits. Moreover, the use of a single resistant source could potentially diminish genetic diversity. Wangshuibai (WSB), an indigenous wheat accession in Jiangsu, China, is highly resistant to FHB and carries QTL for different types of FHB resistance (Lin et al., 2004, 2006; Zhou et al., 2004; Mardi et al., 2005; Yu et al., 2007). Using a recombinant inbred line population, WSB was found to possess type I resistance QTL on chromosomes 3A, 4B (*Fhb4*), and 5A (*Fhb5*), type II resistance QTL on chromosomes 2A, 3B (*Fhb1*), and 6B (*Fhb2*), and type IV resistance QTL on chromosomes 2A, 3B, 4B, and 7D (Lin et al., 2004, 2006; Li et al., 2008; Ma et al., 2008). To speed up utilization of the WSB QTL, *Fhb1* has been cloned (Li et al., 2019b), and *Fhb2, Fhb4*, and *Fhb5* have been mapped to small intervals (Xue et al., 2010b, 2011; Jia et al., 2018).

Evaluation of the QTL effects in different genetic backgrounds is of great importance for marker-assisted breeding. WSB *Fhb1, Fhb4*, and *Fhb5* have been individually validated using near-isogenic lines developed with Mianyang 99–323 as the recurrent parent (Xue et al., 2010a). This study investigated the effects of *Fhb1, Fhb4*, and *Fhb5* pyramiding in five modern Chinese wheat cultivars or lines on FHB resistance and a few major agronomic traits.

## Materials and Methods

### Plant Materials

NMAS022 is a near-isogenic line carrying *Fhb1, Fhb2, Fhb4*, and *Fhb5*, developed through marker-assisted backcross with WSB as the donor parent and FHB-susceptible common wheat line, PH691, as the recurrent parent and is similar to WSB in FHB resistance and PH691 in other traits. The recipients included semi-winter white wheat lines, Bainong418, Bainong4199, Zhoumai27, and 4446, and a semi-winter red wheat cultivar, Chuanmai64. Bainong418 and Bainong4199 were developed by Henan Institute of Science and Technology; Zhoumai27 and Chuanmai64 were released by Zhoukou Academy of Agricultural Sciences and Crop Research Institute of the Sichuan Academy of Agricultural Sciences, respectively.

### Genotyping

Total genomic DNA was extracted from young leaves according to Ma and Sorrells (1995). PCR was performed in Applied Biosystems^TM^ ProFlex^TM^ 96-Well PCR System (ThermoFisher Scientific, MA, USA) following the procedure of Ma et al. (1996). Each 12.5 μl of PCR reaction consisted of 10–30 ng of DNA template, 1 × PCR buffer, 2.5 nmol dNTP, 2 pmol of each primer, 18.75 nmol MgCl_2_, and 0.4 U Taq DNA polymerase.

Marker WGRB619, designed according to the *Fhb1* sequence, was used in *Fhb1* detection (Li et al., 2019b). GWM149 and GWM513 were used in *Fhb4* detection (Xue et al., 2010b). Three markers, including WMC752, BARC180, and MAG9482 (5′-CATGATTGATTCGATGACTATAATATCTT-3′, 5′TCTTTCTCCCGTTGCAATGT-3′), were used for *Fhb5* identification. *Xmag9482* and *Xwmc752* are distal and proximal to *Fhb5* (unpublished data). *Xbarc180* is also proximal to, but further from, *Fhb5* (Xue et al., 2011). The PCR profile was as follows: 94°C for 5 min, followed by 36 cycles of 94°C for 30 s, 52–60°C for 30 s (WGRB619, GWM513, and WMC752 at 60°C; GWM149 at 54°C; MAG9482 and BARC180 at 52°C), and 72°C for 40 s or 2 min (WGRB619), then 72°C for 5 min. WGRB619 PCR products were separated into 1% agarose gels and visualized by ethidium bromide staining. The other PCR products were separated in 8% non-denaturing polyacrylamide gels with acrylamide and bis-acrylamide in 29:1 and visualized by silver staining (Bassam et al., 1991).

### Field Trials

Field trials were conducted in the wheat-growing seasons at Huaiyin Institute of Agricultural Sciences, Huai'an, China, from 2018–2020, using the randomized complete block design with commonly undertaken cultivation practices in wheat production. Two trials, one for type I resistance evaluation and one for type II resistance evaluation, were set up in 2018–2019. Each of the trials consisted of two blocks in which each plot had two 1.5-m rows spaced by 0.25 m. About 25 seeds were planted per row. In 2019–2020, three trials were set up. One trial for type I resistance evaluation and one trial for type II resistance evaluation had two blocks, and one trial for agronomic trait evaluation had three blocks. In the blocks, each plot had 60 seeds planted in a 3-m row and the row spacing was 0.5 m.

### FHB Resistance Evaluation

Type I resistance was evaluated by spraying at anthesis, the mixed conidial suspension (one spore per microliter) of four local virulent strains of *F. graminearum*. About 14 days after the inoculation, 82–100 spikes were selected randomly in each plot and the number of spikes with visible FHB symptoms in at least one of their florets was scored. Percentage of infected spikes (PIS) was used to represent the type I resistance.

Type II resistance was evaluated by single floret inoculation at anthesis. About 10 μl mixed conidial suspension of *F. graminearum* containing 1,000 spores was injected into a flowering floret near the middle of a spike. Twenty spikes were inoculated in each plot, and 10 spikes with the most serious symptom were investigated for the number of diseased spikelets (NDS) and the length of diseased rachides (LDR) at about 18 days after the inoculation to represent the type II resistance.

### Agronomic Trait Evaluation

Anthesis was the time from sowing to more than half of the plants flowering in the plot. Plant height, number of kernels per spike, and number of productive tillers of five plants randomly chosen from the middle of each plot were investigated at physiological maturity and the plot means were used in the analysis. The plant height was the total length of the aboveground part excluding the awn. The number of kernels per spike was counted from the main spikes. The plants located in the middle 1 m of a plot and without inoculations were harvested at maturity for yield and thousand kernel weight (TKW) measurements. TKW was measured after oven-drying.

### Statistical Analysis

A one-way ANOVA was carried out using SPSS Statistics version 25 (IBM, USA). The Tukey test was used in multiple comparisons.

## Results

### Parental Examination With Foreground-Selection Markers

**Figure 1:**
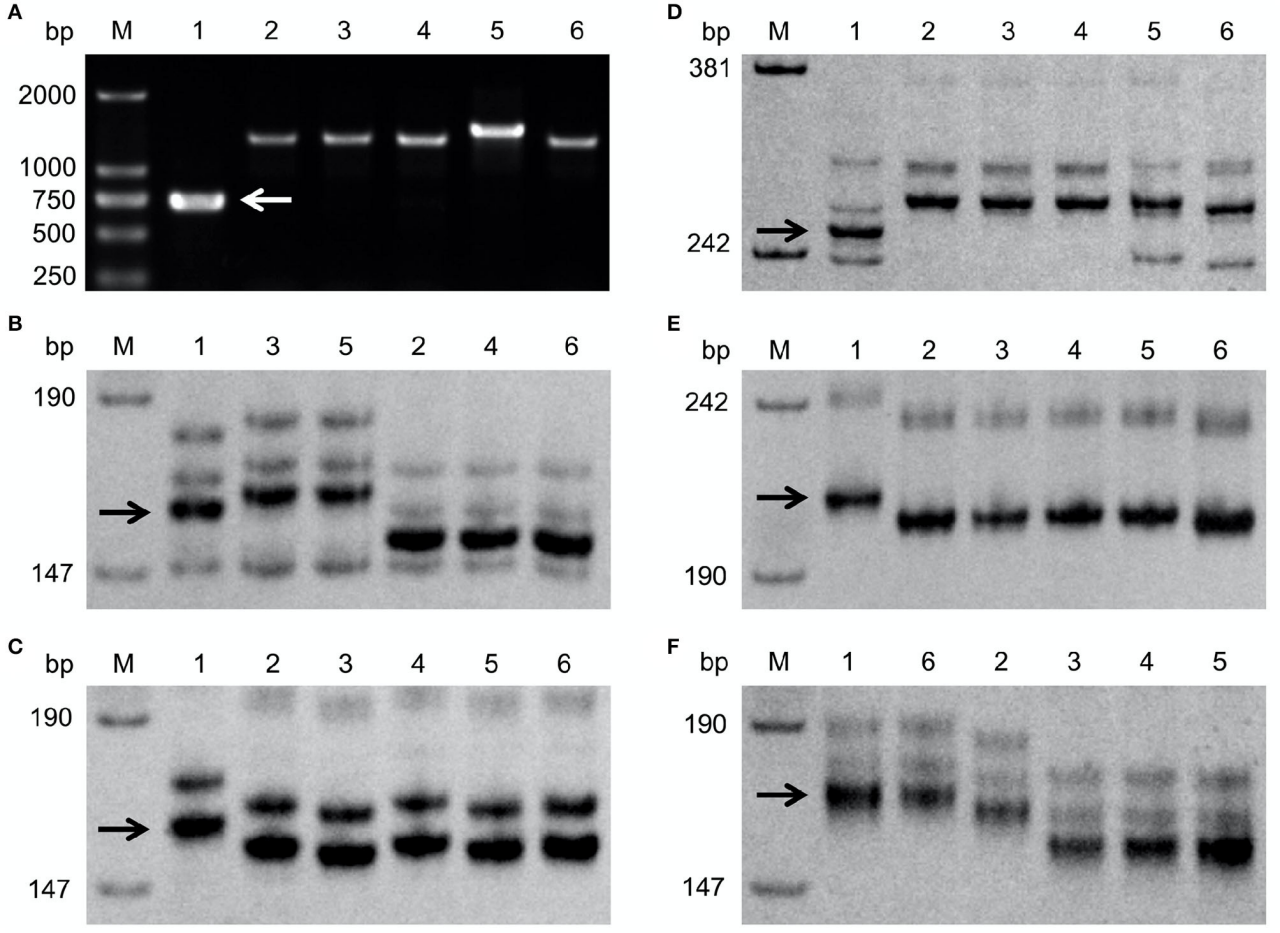
Detection of *Fhb1* by WGRB619 **(A)**, *Fhb4* by GWM149 **(B)** and GWM513 **(C)**, and *Fhb5* by MAG9482 **(D)**, BARC180 **(E)**, and WMC752 **(F)**. The target bands were indicated by arrows. M: the DNA size standard (in bp). 1, 2, 3, 4, 5, and 6: NMAS022, Bainong418, Bainong4199, Zhoumai27, 4446, and Chuanmai 64, respectively.

To obtain markers suitable for selection of *Fhb1, Fhb4*, or *Fhb5* and to find out whether the recipients carry these three QTLs, the five recipient lines for the QTL introgression were examined with markers WGRB619 for *Fhb1*, GWM149 and GWM513 flanking *Fhb4*, and MAG9482 and BARC180 flanking *Fhb5*. None of these lines possess the expected marker alleles (Figure 1), indicating that the five recipients do not carry *Fhb1, Fhb4*, or *Fhb5*. WMC752 detected polymorphism between NMAS022 and among all the recipients but Chuanmai 64 (Figure 1F). It was, therefore, used in the detection of *Fhb5* in introgression into these four cultivars, since *Xwmc752* was located on the same side of *Fhb5* as *Xbarc180* and closer to the QTL peak.

### *Fhb1, Fhb4*, and *Fhb5* Pyramiding

**Figure 2:**
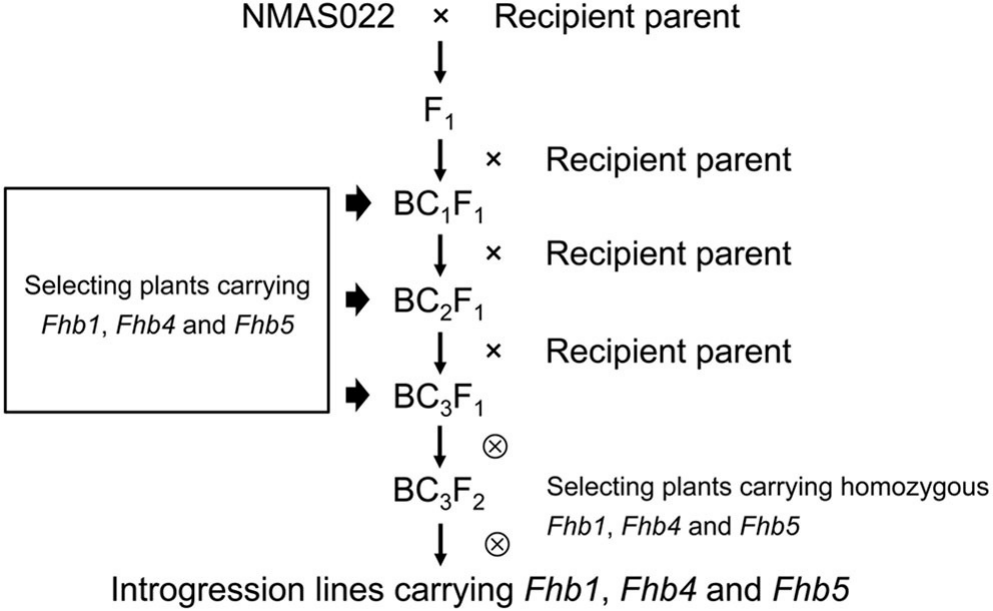
Scheme for *Fhb1, Fhb4*, and *Fhb5* pyramiding.

*Fhb1, Fhb4*, and *Fhb5* were introduced from NMAS022 to Bainong418, Bainong4199, Zhoumai27, 4446, and Chuanmai 64 through three generations of marker-assisted backcross using the recipient lines as recurrent parents (Figure 2). To identify plants carrying the target QTL, an average of 37 plants were examined per generation per cross with the foreground-selection markers (Table 1). In each backcross generation, plants carrying all three target QTLs accounted for 9.4~16.1%, which was in accordance with the expected 1:7 ratio ($\chi_{1:7}^{2}$ = 0~0.37 < $\chi_{0.05,1}^{2}$ = 3.841). To obtain plants homozygous at *Fhb1, Fhb4*, and *Fhb5*, 109–140 BC_3_F_2_ plants from each combination were surveyed with the foreground markers (Table 1). Usually, the plants more similar to the respective recipient parents were chosen for backcrossing or self-seed harvest.

**Table 1:** Population size and the number of plants carrying Wangshuibai (WSB) *Fhb1, Fhb4*, and *Fhb5* in the backcross and F_2_ generations derived from each recipient line.

**Recipient lines**	**Backcross generations**	**Population size**	**No. plants carrying *Fhb1*, *Fhb4*, and *Fhb5***
Bainong418	BC_1_F_1_	31	5
	BC_2_F_1_	39	5
	BC_3_F_1_	30	4
	BC_3_F_2_	122	2
Bainong4199	BC_1_F_1_	40	5
	BC_2_F_1_	42	6
	BC_3_F_1_	36	4
	BC_3_F_2_	140	2
Zhoumai27	BC_1_F_1_	45	5
	BC_2_F_1_	43	6
	BC_3_F_1_	34	4
	BC_3_F_2_	138	1
4446	BC_1_F_1_	36	5
	BC_2_F_1_	39	5
	BC_3_F_1_	41	6
	BC_3_F_2_	115	3
Chuanmai64	BC_1_F_1_	34	5
	BC_2_F_1_	38	5
	BC_3_F_1_	32	3
	BC_3_F_2_	109	2

### FHB Resistance of *Fhb1, Fhb4*, and *Fhb5* Introgression Lines

**Figure 3:**
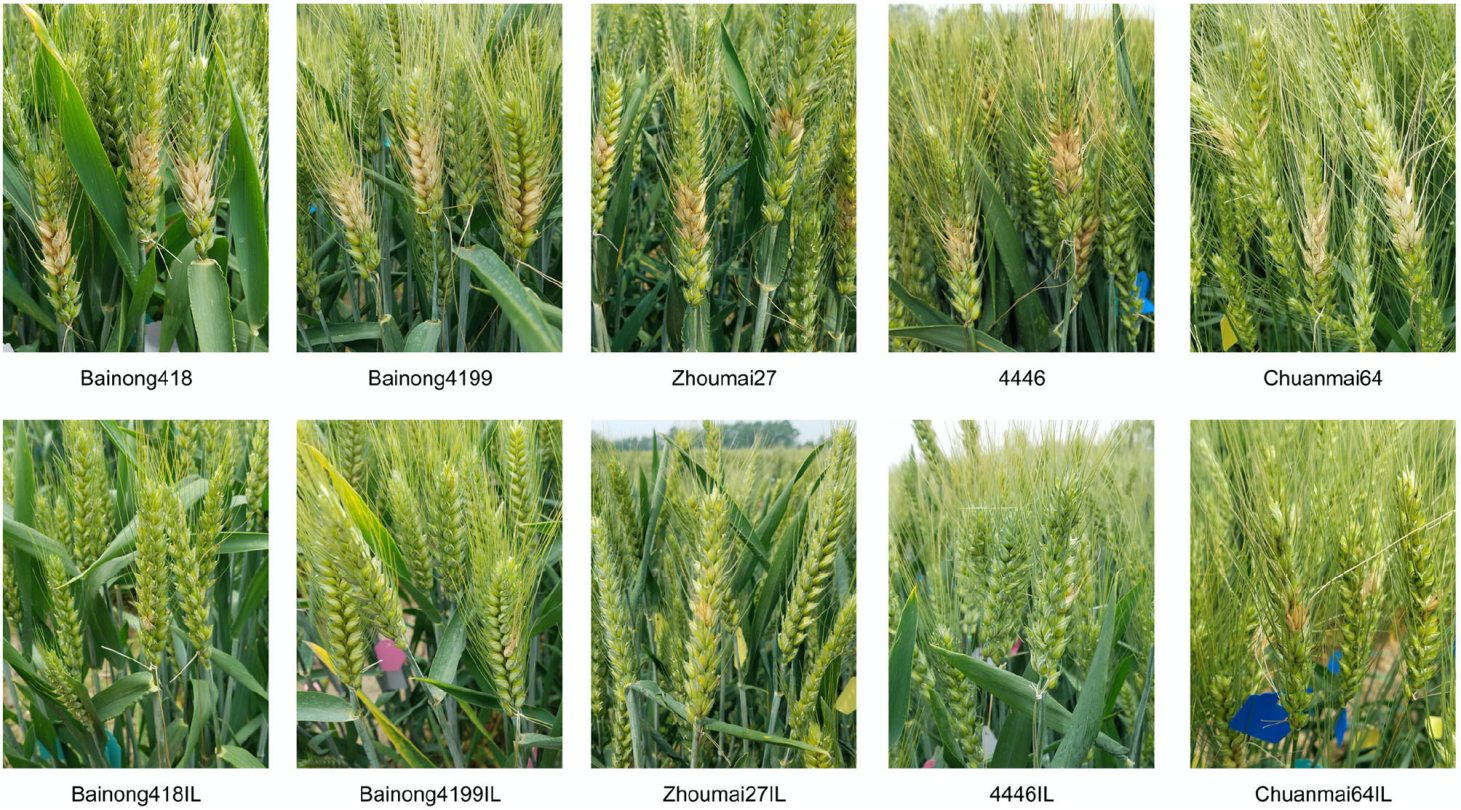
Fusarium head blight (FHB) symptom illustration of the recipients (top) and *Fhb1, Fhb4*, and *Fhb5* introgression lines (bottom). Photos were taken 18 days after single floret inoculation.

The five introgression lines obtained from the BC_3_F_2_ survey and their parents were subjected to type I and type II resistance evaluations in the 2018–2019 season. For both resistance types, all the introgression lines performed significantly better than their recipient parents (Table 2, Figure 3). About 14 days after the spraying inoculation, PIS of the introgression lines was <17%, while that of the recipient parents was higher than 48%. About 18 days after single floret inoculation, the introgression lines had only one diseased spikelet and about 1 cm of LDR, much lower than the recipient parents, which had, on average, 6.1 diseased spikelets and 4.2 cm LDR.

**Table 2:** Percentage of infected spikes (PIS), number of diseased spikelets (NDS), and length of diseased rachides (LDR) (represented as mean ± SD) of the WSB *Fhb1, Fhb4*, and *Fhb5* introgression lines compared with donor parent NMAS022 and the recipient lines.

**2018–2019**				**2019–2020**			
**Lines**	**PIS(%)**	**NDS**	**LDR(cm)**	**Lines**	**PIS(%)**	**NDS**	**LDR(cm)**
NMAS022	13.2 ± 2.4	1.0 ± 0.0	0.3 ± 0.1	NMAS022	9.0 ± 1.4	1.0 ± 0.0	0.2 ± 0.1
Bainong418	51.0 ± 1.4	6.1 ± 0.1	4.4 ± 0.1	Bainong418	38.5 ± 2.1	5.6 ± 0.3	4.1 ± 0.2
Bainong418IL	16.5 ± 2.3^**^	1.0 ± 0.0^**^	1.0 ± 0.1^**^	Bainong418IL-1	9.0 ± 1.4^**^	1.0 ± 0.0^**^	1.0 ± 0.1^**^
				Bainong418IL-2	9.0 ± 2.8^**^	1.0 ± 0.0^**^	0.9 ± 0.0^**^
				Bainong418IL-3	10.0 ± 2.8^**^	1.0 ± 0.0^**^	0.9 ± 0.1^**^
Bainong4199	51.7 ± 3.2	6.4 ± 0.1	4.3 ± 0.1	Bainong4199	37.0 ± 2.8	5.7 ± 0.1	4.0 ± 0.1
Bainong4199IL	16.0 ± 1.6^**^	1.1 ± 0.1^**^	1.1 ± 0.1^**^	Bainong4199IL-1	11.0 ± 1.4^**^	1.0 ± 0.0^**^	0.8 ± 0.1^**^
				Bainong4199IL-2	10.5 ± 2.1^**^	1.0 ± 0.0^**^	0.9 ± 0.1^**^
				Bainong4199IL-3	9.0 ± 2.8^**^	1.0 ± 0.0^**^	0.8 ± 0.1^**^
Zhoumai27	53.9 ± 1.9	5.9 ± 0.2	4.3 ± 0.3	Zhoumai27	39.0 ± 1.4	5.5 ± 0.1	4.0 ± 0.1
Zhoumai27IL	14.3 ± 1.8^**^	1.0 ± 0.0^**^	1.0 ± 0.1^**^	Zhoumai27IL-1	9.5 ± 2.1^**^	1.0 ± 0.0^**^	0.7 ± 0.1^**^
				Zhoumai27IL-2	8.0 ± 1.4^**^	1.0 ± 0.0^**^	0.7 ± 0.1^**^
				Zhoumai27IL-3	7.0 ± 1.4^**^	1.0 ± 0.0^**^	0.8 ± 0.0^**^
4446	50.1 ± 3.0	6.0 ± 0.1	4.2 ± 0.1	4446	40.0 ± 2.8	5.3 ± 0.1	4.1 ± 0.1
4446IL	12.4 ± 2.9^**^	1.1 ± 0.1^**^	1.0 ± 0.1^**^	4446IL-1	9.5 ± 2.1^**^	1.0 ± 0.0^**^	0.9 ± 0.1^**^
				4446IL-2	7.0 ± 1.4^**^	1.0 ± 0.0^**^	0.8 ± 0.1^**^
Chuanmai64	48.7 ± 1.8	5.6 ± 0.1	3.9 ± 0.1	Chuanmai64	35.5 ± 2.1	4.9 ± 0.4	3.8 ± 0.4
Chuanmai64IL	14.1 ± 1.6^**^	1.0 ± 0.0^**^	0.9 ± 0.1^**^	Chuanmai64IL-1	10.0 ± 1.4^**^	1.0 ± 0.0^**^	0.8 ± 0.1^**^
				Chuanmai64IL-2	8.0 ± 1.4^**^	1.0 ± 0.0^**^	0.8 ± 0.0^**^
				Chuanmai64IL-3	9.0 ± 0.0^**^	1.0 ± 0.0^**^	0.9 ± 0.1^**^

** *indicate significance at P = 0.01, compared with the respective recipients. Comparison of the introgression lines with NMAS022 revealed significant differences only for LDR, which was not shown in the table*.

It was noted in the field that the overall morphology of these introgression lines was similar to their recipient parents, but variations in some traits, such as plant height and spike shape, still existed. Thus, two to three plants were selected from each line with these variations in mind for further evaluation of both FHB resistance and agronomic traits in the 2019–2020 season. It was shown that all 14 selected lines still had significantly less NDS and LDR than the recipient parents (Table 2), indicating the stability of the resistance conferred by the three QTLs. All the introgression lines were similar to NMAS022 in terms of PIS and NDS but had longer diseased rachides (Table 2). These results indicated that the introgression of *Fhb1, Fhb4*, and *Fhb5* led to a type I resistance level comparative to the QTL donor parent and a significantly improved type II resistance.

### Agronomic Performance of the *Fhb1, Fhb4*, and *Fhb5* Introgression Lines

To determine the effects of *Fhb1, Fhb4*, and *Fhb5* pyramiding on agronomic performance, six traits, namely, anthesis, plant height, number of kernels per spike, number of productive tillers, TKW, and 0.5-m^2^ yield, were investigated. NMAS022 was different from all the recipient lines in most of the investigated traits (Table 3). In comparison with the respective recipient parents, the introgression lines were similar in anthesis, and the introgression line 4446IL-1 was the only one showing significant variation in the number of productive tillers. As to the remaining four traits, some introgression lines were similar to the recipient parents, some showed positive changes, and some varied negatively (Table 3). It was, therefore, concluded that it was not the *Fhb1, Fhb4*, and *Fhb5* pyramiding but the variations of genetic composition that conditioned the agronomic trait variations. Interestingly, the 0.5-m^2^ yield of all the introgression lines was similar to or even significantly higher than the respective recipient parents.

**Table 3:** Agronomic traits (represented as mean ± SD) of the WSB *Fhb1, Fhb4*, and *Fhb5* introgression lines and the parents.

**Lines**	**Anthesis (day)**	**Plant height (cm)**	**No. productive tillers**	**No. kernels per spike**	**TKW (g)**	**0.5-m^**2**^ yield (g)**
NMAS022	183 ± 0.0	145.5 ± 1.7	12.3 ± 0.6	51.7 ± 0.8	48.3 ± 0.3	306.7 ± 5.8
Bainong418	176 ± 0.0	84.5 ± 0.8	12.1 ± 0.8	57.9 ± 1.0	54.7 ± 0.7	363.3 ± 5.8
Bainong418IL-1	176 ± 0.0	85.9 ± 1.0	13.3 ± 0.4	58.5 ± 0.8	49.9 ± 0.5**	366.7 ± 5.8
Bainong418IL-2	175 ± 0.0	87.3 ± 0.5**	12.6 ± 0.5	57.5 ± 1.5	52.8 ± 1.0*	360.0 ± 0.0
Bainong418IL-3	175 ± 0.0	90.5 ± 0.4**	12.8 ± 0.7	56.7 ± 0.9	53.0 ± 0.6	366.7 ± 5.8
Bainong4199	176 ± 0.0	79.3 ± 0.9	12.1 ± 0.6	59.1 ± 1.1	44.3 ± 0.8	380.0 ± 0.0
Bainong4199IL-1	175 ± 0.0	80.9 ± 0.4	13.1 ± 0.4	55.1 ± 0.6**	45.0 ± 0.6	383.3 ± 5.8
Bainong4199IL-2	176 ± 0.0	83.5 ± 0.8**	11.4 ± 0.6	58.2 ± 0.9	50.9 ± 0.6**	393.3 ± 5.8*
Bainong4199IL-3	176 ± 0.0	83.9 ± 0.8**	11.7 ± 0.5	53.9 ± 0.5**	48.8 ± 0.8**	376.7 ± 5.8
Zhoumai27	178 ± 0.0	81.1 ± 0.8	11.1 ± 0.6	68.3 ± 0.6	48.0 ± 0.7	376.7 ± 5.8
Zhoumai27IL-1	177 ± 0.0	89.8 ± 0.8**	12.2 ± 0.4	63.6 ± 0.6**	52.4 ± 0.7**	406.7 ± 5.8**
Zhoumai27IL-2	179 ± 0.0	81.5 ± 0.8	11.4 ± 0.7	70.3 ± 0.6*	45.4 ± 0.8*	373.3 ± 5.8
Zhoumai27IL-3	177 ± 0.0	84.0 ± 0.7**	12.0 ± 0.7	68.3 ± 1.0	43.8 ± 0.7**	380.0 ± 0.0
4446	175 ± 0.0	85.5 ± 1.0	8.9 ± 0.5	57.7 ± 1.2	57.2 ± 0.8	333.3 ± 5.8
4446IL-1	175 ± 0.0	87.4 ± 1.2	10.9 ± 0.8*	58.3 ± 1.3	57.3 ± 0.4	360.0 ± 10.0**
4446IL-2	176 ± 0.0	85.1 ± 0.8	9.3 ± 0.5	58.2 ± 0.9	54.3 ± 0.6**	340.0 ± 0.0
Chuanmai64	175 ± 0.0	104.1 ± 0.9	11.7 ± 0.6	56.3 ± 0.9	43.1 ± 0.7	423.3 ± 5.8
Chuanmai64IL-1	175 ± 0.0	120.5 ± 0.3**	11.9 ± 0.5	55.9 ± 1.3	45.0 ± 0.7	420.0 ± 0.0
Chuanmai64IL-2	175 ± 0.0	122.1 ± 0.4**	11.7 ± 0.8	56.3 ± 0.6	42.6 ± 1.1	426.7 ± 5.8
Chuanmai64IL-3	175 ± 0.0	104.3 ± 0.4	12.3 ± 0.4	56.2 ± 0.5	39.4 ± 0.6**	416.7 ± 5.8

*, ** indicate significance at P = 0.05 and P = 0.01, respectively, compared with the respective recipients. All the recipient lines were significantly different from NMAS022 in anthesis, plant height, number of kernels per spike, and 0.5-m^2^ yield (P = 0.01), and NMAS022 had more productive tillers than line 4446 and higher TKW than all the recipients but Zhoumai27, which were not shown in the table.

## Discussion

In this study, the FHB resistance QTLs, *Fhb1, Fhb4*, and *Fhb5*, from WSB were simultaneously introduced into five modern wheat cultivars or lines adapting to different wheat-growing areas using a marker-assisted backcross strategy. A 2-year FHB resistance evaluation indicated that *Fhb1, Fhb4*, and *Fhb5* pyramiding significantly improved both type I and type II resistances in all backgrounds without exception. Due to the significant correlation of *Fhb1, Fhb4*, and *Fhb5* intervals with mycotoxin DON accumulation (Somers et al., 2003; Jiang et al., 2007a,b; Bonin and Kolb, 2009; Jayatilake et al., 2011; Szabó-Hevér et al., 2014), the developed introgression lines are expected to reduce the kernel DON level too.

*Fhb1* improves only type II resistance, and *Fhb4* and *Fhb5* enhance only type I resistance (Lin et al., 2004, 2006; Ma et al., 2008; Xue et al., 2010a). Compared with the introduction of a single QTL, pyramiding of QTLs for different types of FHB resistance is more effective against the disease, as illustrated in FHB resistance improvement of AK58 by Xu et al. (2017), and should be promoted in breeding programs due to the lack of genes conferring immunity to FHB. It was noted that the introgression lines had longer diseased rachides after point inoculation than NMAS022 that carries *Fhb2* as well as *Fhb1, Fhb4*, and *Fhb5*, implying that the introgression of *Fhb2* could further improve the FHB resistance.

The total disease index was not investigated in this study because of the limitation of the experiments; however, the obtained results were still telling since the local pathogen pressure imposed by artificial inoculation in the resistance evaluation was far greater than that imposed by natural inoculation. Based on the NDS obtained after single floret inoculation and the PIS obtained after spraying inoculation, the *Fhb1, Fhb4*, and *Fhb5* pyramiding raised the FHB resistance level by 95% and made the introgression lines highly resistant to FHB. The QTL pyramiding effects are, however, still in dispute, as shown by Brar et al. (2019a), who introduced *Fhb1, Fhb2*, and *Fhb5* from Sumai 3 into two hard red spring wheat cultivars from Canada, and by Salameh et al. (2011), who made a similar attempt in European winter wheat. We reasoned that the discrepancy could be due to the small effect of *Fhb2* (unpublished data), different trial conditions and resistance evaluation methods, and the genetic backgrounds.

Wheat breeders often find it difficult to obtain plants with satisfied agronomic performance and a high level of FHB resistance in conventional breeding using Sumai 3 as a parent, which prompts deliberation on whether the FHB resistance genes have deleterious effects on agronomic traits. Indeed, the *Fhb4* interval showed association with plant height (Jia et al., 2013), and the *Fhb5* interval was related to plant height and grain weight (Huang et al., 2004, 2015; Jia et al., 2013; Steiner et al., 2019). In a few studies, the introduction of *Fhb4* interval resulted in plant height increase (McCartney et al., 2007; Xue et al., 2010a), and the introduction of *Fhb5* interval led to lower TKW and a slight increase in plant height (Brar et al., 2019b). We demonstrated, using multiple parental combinations, that these associations can be broken through selection, particularly with the help of suitable markers. In terms of yield performance, the *Fhb1, Fhb4, and Fhb5* introgression lines were as good as the recipient parents. The yield of introgression line 4446IL-1 even increased up to 8%. These results suggested that the *Fhb1, Fhb4*, and *Fhb5* pyramiding was not in conflict with agronomic trait improvement.

Marker-assisted selection has displayed the potential in improving FHB resistance breeding efficiency. In addition to breaking up unfavorable linkage drags, MAS can also speed up the breeding process (Xue et al., 2010a; Brar et al., 2019a). Taking *Fhb1, Fhb4*, and *Fhb5*, which are located on different chromosomes, as an example, the plants carrying all three QTLs theoretically account for one-eighth in each backcross. Therefore, the probability of obtaining such a plant is more than 98% when more than 30 BCF_1_ plants are surveyed. Enlarging the backcross population size manageably, together with marker-assisted background selection, could greatly accelerate the QTL introgression (Xue et al., 2010a; Huang et al., 2015). This study showed again the usefulness and effectiveness of the *Fhb1* functional marker and the closely-linked *Fhb4* and *Fhb5* flanking markers.

The recipient parents used in this study were all newly bred cultivars or lines and represented different ecological types. The obtained introgression lines could not only be used as breeding parents but also have the potential to be directly deployed in production.

## Data Availability Statement

The original contributions presented in the study are included in the article/supplementary material, further inquiries can be directed to the corresponding author/s.

## Author Contributions

YZ conducted experiments, data analysis, and prepared the draft. HM, LH, FD, and CR participated in genotyping and material development. ZY, YD, and ZG contributed to phenotyping. HJ, GL, and ZK contributed to project implementation. ZM designed the project and reviewed the article. All the authors have read and approved the final manuscript.

## Conflict of Interest

The authors declare that the research was conducted in the absence of any commercial or financial relationships that could be construed as a potential conflict of interest.
